# A Phenotyping of Diastolic Function by Machine Learning Improves Prediction of Clinical Outcomes in Heart Failure

**DOI:** 10.3389/fcvm.2021.755109

**Published:** 2021-12-23

**Authors:** Haruka Kameshima, Tokuhisa Uejima, Alan G. Fraser, Lisa Takahashi, Junyi Cho, Shinya Suzuki, Yuko Kato, Junji Yajima, Takeshi Yamashita

**Affiliations:** ^1^The Cardiovascular Institute Hospital, Tokyo, Japan; ^2^School of Medicine, Cardiff University, Cardiff, United Kingdom; ^3^Tokyo Medical University Hospital, Tokyo, Japan

**Keywords:** machine learning, echocardiogram classification, diastolic function, heart failure, prognostication factor

## Abstract

**Background:** Discriminating between different patterns of diastolic dysfunction in heart failure (HF) is still challenging. We tested the hypothesis that an unsupervised machine learning algorithm would detect heterogeneity in diastolic function and improve risk stratification compared with recommended consensus criteria.

**Methods:** This study included 279 consecutive patients aged 24–97 years old with clinically stable HF referred for echocardiographic assessment, in whom diastolic variables were measured according to the current guidelines. Cluster analysis was undertaken to identify homogeneous groups of patients with similar profiles of the variables. Sequential Cox models were used to compare cluster-based classification with guidelines-based classification for predicting clinical outcomes. The primary endpoint was hospitalization for worsening HF.

**Results:** The analysis identified three clusters with distinct properties of diastolic function that shared similarities with guidelines-based classification. The clusters were associated with brain natriuretic peptide level (*p* < 0.001), hemoglobin concentration (*p* = 0.017) and estimated glomerular filtration rate (*p* = 0.001). During a mean follow-up period of 2.6 ± 2.0 years, 62 patients (22%) experienced the primary endpoint. Cluster-based classification predicted events with a hazard ratio 1.68 (*p* = 0.019) that was independent from and incremental to the Meta-analysis Global Group in Chronic Heart Failure (MAGGIC) risk score for HF, and from left ventricular end-diastolic volume and global longitudinal strain, whereas guidelines-based classification did not retain its independent prognostic value (hazard ratio = 1.25, *p* = 0.202).

**Conclusion:** Machine learning can identify patterns of diastolic function that better stratify the risk for decompensation than the current consensus recommendations in HF. Integrating this data-driven phenotyping may help in refining prognostication and optimizing treatment.

## Introduction

Left ventricular (LV) diastolic dysfunction is a key pathophysiological feature of heart failure (HF) and its assessment plays an important role in diagnosing, monitoring and prognosticating HF. It appears early in the natural course of various types of cardiovascular diseases and once severe, diastolic dysfunction is associated with elevated left atrial pressure (1, 2). The clinical diagnosis of HF requires not only the presence of symptoms and/or signs of HF, but also objective evidence of cardiac structural or functional abnormalities, including LV diastolic dysfunction, especially when LV ejection fraction (LVEF) is preserved. LV diastolic dysfunction also predicts adverse outcomes in HF, as demonstrated in a number of large-scale cohort studies (1, 3–5).

In clinical practice, LV diastolic function is assessed using echocardiography by measuring multiple variables, for example from transmitral flow and mitral annular velocity profiles. Each variable reflects a different physiological aspect of LV filling, and all are inter-related in a complex manner (2). Standard criteria do not always change linearly with the elevation of LV filling pressure (6). No single parameter by itself is robust enough to be used for diagnosing diastolic dysfunction, so it is recommended that all relevant parameters should be taken into account when grading diastolic dysfunction. Consensus diagnostic recommendations propose decision-tree algorithms which have been constructed based on expert consensus and theoretical considerations rather than on clinical evidence (7). The utility of the updated consensus recommendations requires further investigation.

Machine learning is a method for analyzing data that, unlike traditional statistics, can deal with complex datasets with multivariable non-linear interactions. It constructs analytical models to extract insights, patterns and relationships that can be used for decision-making. In cardiovascular medicine, machine learning has identified new clinical phenotypes, predicted responses to treatment, and improved prognostication (8–10). Accordingly, we tested the hypothesis that applying cluster analysis would detect heterogeneity in diastolic function and improve risk stratification in a HF population.

## Materials and Methods

### Study Population

**Figure 1:**
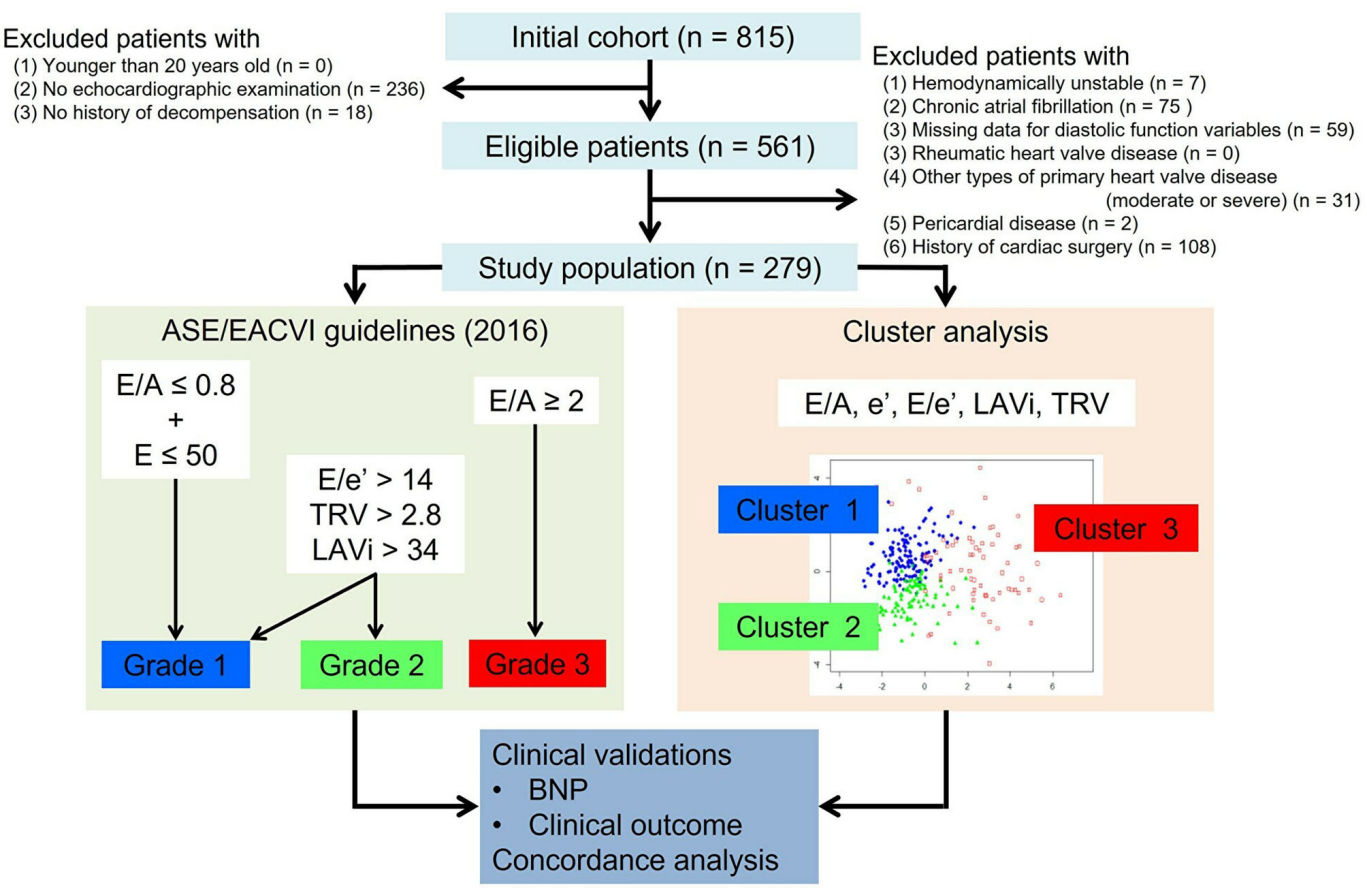
Study design.

From the HF database of the Cardiovascular Institute, Japan (Shinken database, registered in University Hospital Medical Information Network, ID000008598), we retrospectively identified a consecutive series of 815 patients with clinically stable HF. Patients were eligible for this study if they were older than 20 years at the time of the index echocardiographic examination, and if they had been referred to echocardiography for hemodynamic assessment between February 2010 and August 2018, and if they had a history of previous hospitalization for acute decompensated HF with symptoms sufficient to warrant hospitalization and for which intravenous therapy was required. In total 561 patients fulfilled these criteria, but those were excluded who had any of the following: (1) being treated with intravenous therapy at the index examination, (2) atrial fibrillation at the index examination (patients with paroxysmal atrial fibrillation who were in sinus rhythm at the index examination were included), (3) any missing data for diastolic variables, (4) rheumatic heart valve disease, (5) any other types of primary heart valve disease more than moderate, (6) pericardial disease and (7) history of cardiac surgery (Figure 1). This exclusion left 279 patients as the study population.

This study was approved by the institutional review board. All the patients gave written informed consent when registered in the hospital database.

### LV Chamber Quantification and Guidelines-Based Classification of Diastolic Function

Comprehensive echocardiographic examination was performed, using commercially available ultrasound machines with a 2.5 MHz sector transducer (Vivid E95 and E9, General Electric Company; iE33 and Epic7, Phillips; Artida, Cannon; Prosound alpha10, Hitachi). LV end-diastolic and end-systolic volumes and LVEF were calculated by the disk summation method. LV mass was calculated as recommended and normalized by body surface area (LVMi). LV global longitudinal and circumferential strains were measured using Image Arena (TOMTEC Imaging Systems GmbH, Germany). Left atrial volume was also measured by the disk summation method and normalized by body surface area (LAVi). Pulse-wave Doppler tracings at the tip of the mitral valve leaflets were recorded, and early to late diastolic transmitral velocity ratio (E/A) was calculated. Mitral annular velocities were recorded using pulsed tissue Doppler from the base of the septum in an apical 4-chamber view, to evaluate LV longitudinal function (s', e' and a'). Left atrial pressure was estimated from the ratio of transmitral E velocity to early diastolic mitral annular velocity (E/e'). A continuous-wave Doppler tracing of tricuspid regurgitation (TRV) was recorded to assess pulmonary hypertension. Right ventricular end-diastolic area and fractional area change were measured in an apical 4-chamber view to derive right ventricular size and systolic function.

The current American Society of Echocardiography/European Association of Cardiovascular Imaging consensus recommendations proposed two different algorithms for assessing diastolic function (7). In this study, diastolic function was graded using the algorithm for patients with reduced LVEF and/or myocardial disease. This algorithm uses 4 variables: E/A, E/e', LAVi, and TRV and classifies patients into grade 1–3 diastolic dysfunction. Because lateral e' velocity was not available, we employed septal E/e'>15, instead of average E/e'>14 for grading (7). Because it was an inclusion criterion for this study that every patient had a complete dataset of diastolic variables, no patient was graded as indeterminate.

### Cluster Analysis

Model-based cluster analysis, an unsupervised machine learning algorithm, was performed using mclust package in R (version 3.5.1, Vienna, Austria). This assumes that data points within a cluster are normally distributed, and it finds the mixture of multi-dimensional Gaussian probability distributions that best models the input dataset (11).

The analysis was applied in order to group the study population into clusters with distinct diastolic function properties. The diastolic variables used for cluster modeling were chosen based on their inclusion in current consensus recommendations (E/A, e', E/e', LAVi and TRV) (7). The degree of correlations between these variables were assessed by partial correlation analysis. All these variables were standardized to a mean = 0 and a standard deviation = 1 so that they were equally weighted in the analysis. The optimal number of clusters was selected based on Bayesian information criterion. The model parameters were estimated using expectation-maximization algorithm. The patients were assigned to a cluster where their posterior probability of membership was the highest. The clusters were numbered from low to high average values of E/e' and LAVi.

### Clinical Relevance

The learned clusters were characterized by comparing clinical and echocardiographic variables. These variables, including demographics, vital signs, cardiac risk factors, HF symptoms, time since the first diagnosis of HF, drug treatment, and laboratory data such as hemoglobin level and estimated glomerular filtration rate (eGFR), which had all been recorded within 3 months of the index examination, were collected from the hospital database. Brain natriuretic peptide (BNP) measurement was available in 193 (70%) patients. The Meta-analysis Global Group in Chronic Heart Failure (MAGGIC) score, an established clinical risk score for HF, was calculated from all relevant variables (12).

Whether or not the learned clusters reasonably captured diastolic phenotypes was tested by studying the associations of BNP level and clinical outcomes with the clusters. The outcome data were obtained from the hospital database which integrates events documented in the hospital medical records and those recorded through an annual postal survey. Patients were censored when they stopped visiting the hospital or responding to the postal survey. Those who continued to attend and remained free from events at 5 years were automatically censored then. The primary endpoint was hospitalization for worsening HF. The secondary endpoint was a composite of cardiovascular death and hospitalization for worsening HF. The cardiovascular death was ascertained from medical records of the patients and from direct contact with local physicians. Sudden death was considered as cardiovascular death in this study.

### Discordance Between Cluster-Based and Guidelines-Based Classifications

We did not expect full agreement between the two classifications and would rather highlight discordance where cluster-based classification could help further risk stratification. Principal component analysis was performed on the same five diastolic variables as used for the clustering, to plot and compare the patterns of grade and cluster distribution. A contingency table was created to identify where cluster-based classification was the most discordant with guidelines-based classification. BNP level and clinical outcomes were compared among the clusters in a subgroup of patients with discordant classifications.

### Statistical Analysis

Categorical variables were expressed as number and percentage and were compared using chi-square test. Continuous variables were expressed as mean ± standard deviation and were compared using analysis of variance if the variables were normally distributed. If they were not found to be normally distributed, they were expressed as median (25–75th percentile) and were compared using Kruskal-Wallis test. Survival curves were estimated separately for cluster-based and guidelines-based classifications, using the Kaplan-Meier method, and compared using a log-rank test. Sequential Cox models were used to compare cluster-based and guidelines-based classifications. A baseline model was constructed by entering MAGGIC score and LV end-diastolic volume (LVEDV) and LV global longitudinal strain (LVGLS). We did not add LVEF in the model because it is already included in the MAGGIC score. Nested Cox models with separate addition of cluster-based and guidelines-based classification to the baseline model were constructed. The increase in predictive power after the addition of the classification variables was assessed by the change in overall model χ (2). Concordance between the two diastolic function classifications was assessed using Cohen's kappa statistic and Kendall's correlation coefficient. *P* value < 0.05 was considered as statistically significant. All statistical analyses were performed using IBM SPSS statistics version 19 (International Business Machines Corporation, Illinois, United States of America).

## Results

### Study Population

**Figure 2:**
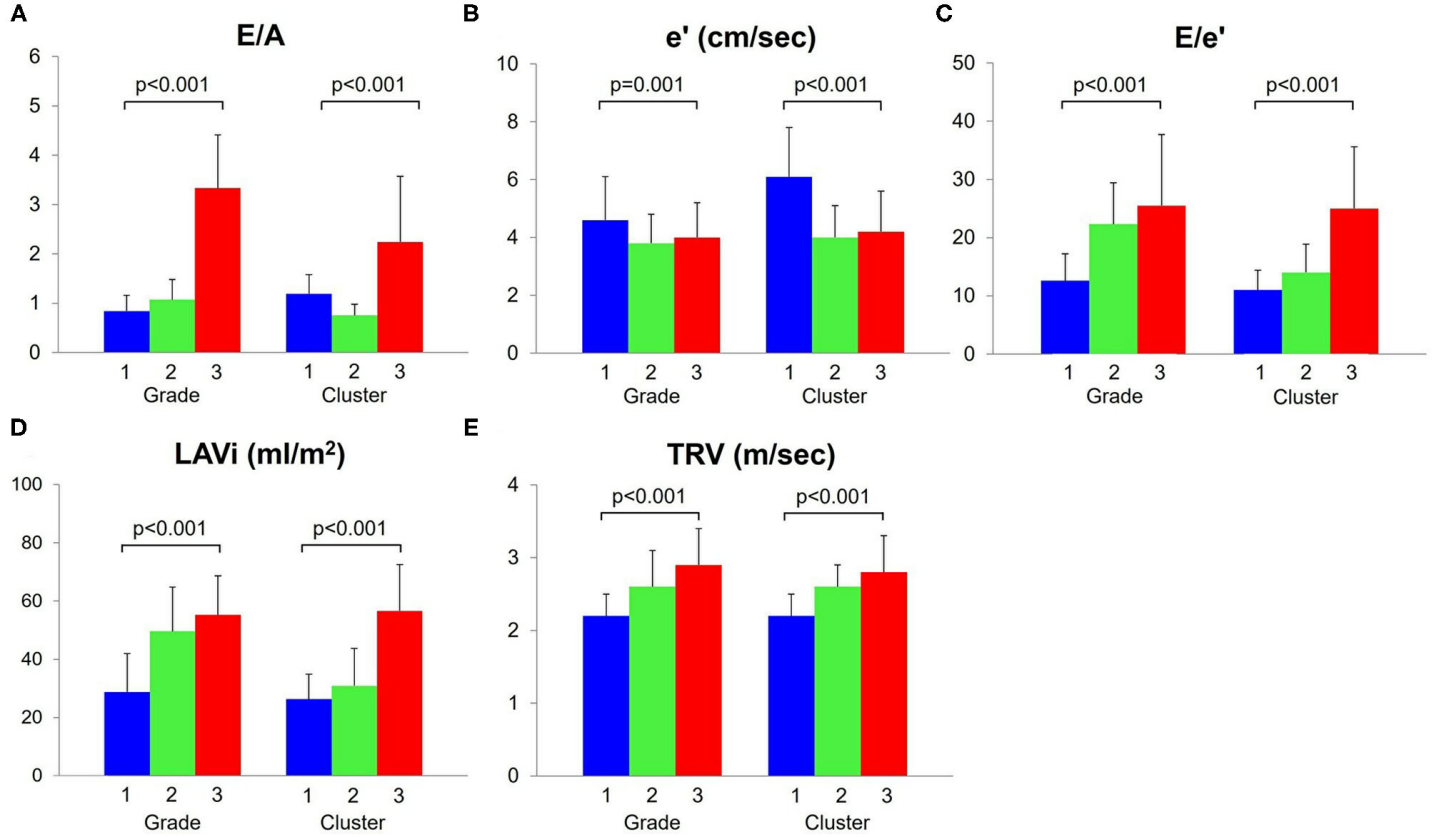
Comparisons of diastolic function variables. These five variables **(A–E)** were used for cluster analysis. The e' **(B)** decreased and E/e' **(C)**, LAVi **(D)** and TRV **(E)** increased, as diastolic function worsened (grade/cluster number increased). LAVi, left atrium volume index; TRV, tricuspid regurgitation velocity.

The comparisons of diastolic variables across the grades are illustrated in Figure 2 and the baseline characteristics are summarized in Table 1. A majority of the subjects (70%) were diagnosed as grade 1 diastolic dysfunction according to the current consensus recommendations. By definition, E/A (*p* < 0.001), E/e' (*p* < 0.001), LAVi (*p* < 0.001) and TRV (*p* < 0.001) progressively increased from grade 1 to 3. The e' (*p* = 0.001) was higher in grade 1 than in grade 2 and 3. Patients with grade 2 diastolic dysfunction were older (*p* < 0.001), more often women (*p* < 0.001) and more often had comorbidities such as hypertension (*p* = 0.001), diabetes (*p* = 0.016) than those with the other grades; heart failure with preserved ejection fraction (HFpEF, LVEF ≥ 50%) accounted for more than half in this subgroup. In comparison, patients with grade 3 diastolic dysfunction had lower blood pressure (*p* < 0.001) and were more often prescribed diuretics (*p* = 0.026). Heart failure with reduced ejection fraction (HFrEF, LVEF < 40%) associated with was prevalent in this subgroup; global circumferential strain was lower (*p* = 0.016) than in the other grades, although LVGLS was not different (*p* = 0.112); right ventricular function was also reduced (*p* < 0.001).

**Table 1:** Baseline characteristics of the study population.

		**Diastolic function grade**			
	**Total** **(*n* = 279)**	**Grade 1** **(*n* = 188)**	**Grade 2** **(*n* = 59)**	**Grade 3** **(*n* = 32)**	***P*** **value**
Age, years	68 ± 15	65 ± 14	76 ± 13	65 ± 14	<0.001
Male gender, *n* (%)	193 (69)	141 (75)	27 (46)	25 (78)	<0.001
BMI, kg/m^2^	23.9 ± 5.1	24.2 ± 5.2	24.0 ± 5.0	22.2 ± 4.1	0.150
SBP, mmHg	120 ± 19	120 ± 18	126 ± 20	109 ± 19	<0.001
HR, beats/min	67 ± 12	67 ± 11	70 ± 14	67 ± 12	0.321
**Comorbidities**					
Hypertension, *n* (%)	183 (66)	111 (59)	50 (85)	22 (69)	0.001
Diabetes, *n* (%)	103 (37)	60 (32)	31 (53)	12 (38)	0.016
**Underlying heart disease**					
CAD, *n* (%)	96 (34)	57 (30)	26 (44)	13 (41)	0.112
Paroxysmal AF, *n* (%)	91 (33)	62 (33)	16 (27)	13 (41)	0.416
HF duration, years	1.4 ± 2.7	1.4 ± 2.8	1.5 ± 2.4	1.5 ± 2.5	0.319
MAGGIC score	22 ± 8	20 ± 8	26 ± 8	24 ± 7	<0.001
**Medications**					
ACEi, *n* (%)	86 (31)	60 (32)	16 (27)	10 (31)	0.784
ARB, *n* (%)	142 (51)	96 (51)	31 (53)	15 (47)	0.872
β-blocker, *n* (%)	211 (76)	144 (77)	39 (66)	28 (88)	0.066
Loop diuretics, *n* (%)	197 (71)	124 (66)	45 (76)	28 (88)	0.026
MRA, *n* (%)	114 (41)	74 (39)	24 (41)	16 (50)	0.527
**Devices**					
Pacemaker, *n* (%)	19 (7)	10 (5)	8 (14)	1 (3)	0.061
CRT, *n* (%)	3 (1)	3 (2)	0 (0)	0 (0)	0.480
ICD, *n* (%)	10 (4)	7 (4)	2 (3)	1 (3)	0.982
**Laboratory**					
Albumin, g/dl	3.9 ± 0.5	4.0 ± 0.5	3.8 ± 0.4	3.7 ± 0.4	<0.001
Hemoglobin, g/dl	13.2 ± 2.0	13.5 ± 1.9	12.0 ± 1.9	13.4 ± 2.5	<0.001
Creatinine, mol/l	97 ± 39	95 ± 42	108 ± 39	93 ± 27	0.036
eGFR, ml/min/1.73 m^2^	55.9 ± 20.6	59.4 ± 20.8	43.9 ± 17.4	57.3 ± 17.1	<0.001
BNP, pg/ml	233 ± 395	124 ± 159	346 ± 346	700 ± 894	<0.001
**Electrocardiography**					
QRS duration, msec	113 ± 35	112 ± 37	119 ± 32	107 ± 23	0.226
**Echocardiography**					
LVEDV, ml	135 ± 66	133 ± 63	130 ± 78	161 ± 57	0.069
LVMi, g/m^2^	157 ± 55	150 ± 47	173 ± 60	175 ± 76	0.002
RWT	0.36 ± 0.11	0.36 ± 0.10	0.39 ± 0.13	0.31 ± 0.12	0.005
LVEF, %	49 ± 17	50 ± 17	50 ± 17	40 ± 13	0.009
<40%, *n* (%)	93 (33)	58 (31)	19 (32)	16 (50)	0.046
40 - 49%, *n* (%)	52 (19)	37 (20)	7 (12)	9 (25)	
≥ 50%, *n* (%)	134 (48)	93 (50)	33 (56)	8 (25)	
GLS, %	−10.9 ± 4.7	−11.2 ± 4.5	−10.7 ± 4.8	−9.3 ± 6.0	0.112
GCS, %	−16.8 ± 8.3	−17.3 ± 8.3	−17.0 ± 8.3	−12.8 ± 6.8	0.016
s', cm/sec	4.9 ± 1.6	5.1 ± 1.6	4.6 ± 1.4	4.0 ± 1.5	<0.001
e', cm/sec	4.3 ± 1.4	4.6 ± 1.5	3.8 ± 1.0	4.0 ± 1.2	0.001
a', cm/sec	6.3 ± 2.2	7.0 ± 1.9	5.7 ± 1.8	3.5 ± 1.0	<0.001
E/A	1.18 ± 0.92	0.84 ± 0.32	1.07 ± 0.41	3.33 ± 1.08	<0.001
E, cm/s	63 ± 23	53 ± 15	81 ± 21	91 ± 24	<0.001
E/e'	16.1 ± 8.3	12.6 ± 4.6	22.3 ± 7.1	25.5 ± 12.2	<0.001
LAVi, ml/m^2^	36.2 ± 17.4	28.8 ± 13.1	49.6 ± 15.2	55.2 ± 13.5	<0.001
TRV, m/sec	2.3 ± 0.4	2.2 ± 0.3	2.6 ± 0.5	2.9 ± 0.5	<0.001
RVEDA, cm^2^	16.0 ± 5.5	15.7 ± 5.0	15.8 ± 6.7	18.5 ± 5.1	0.024
RVFAC, %	48 ± 14	49 ± 13	51 ± 13	37 ± 16	<0.001

*BMI, body mass index; SBP, systolic blood pressure; HR, heart rate; CAD, coronary artery disease; AF, atrial fibrillation; HF, heart failure; MAGGIC, Meta-analysis Global Group in Chronic Heart Failure; ACEi, angiotensin-converting enzyme inhibitor; ARB, angiotensin receptor blocker; MRA, mineralocorticoid receptor antagonist; CRT, cardiac resynchronization therapy; ICD, implantable cardioverter defibrillator; eGFR, estimate glomerular filtration rate; BNP, brain natriuretic peptide; LVEDV, left ventricular end-diastolic volume; LVMi, left ventricular mass index; RWT, relative wall thickness; LVEF, left ventricular ejection fraction; GLS, left ventricular global longitudinal strain; GCS, left ventricular global circumferential strain; LAVi, left atrium volume index; TRV, tricuspid regurgitation velocity; RVEDA, right ventricular end-diastolic area; RVFAC, right ventricular fractional area change*.

### Clustering

**Figure 3:**
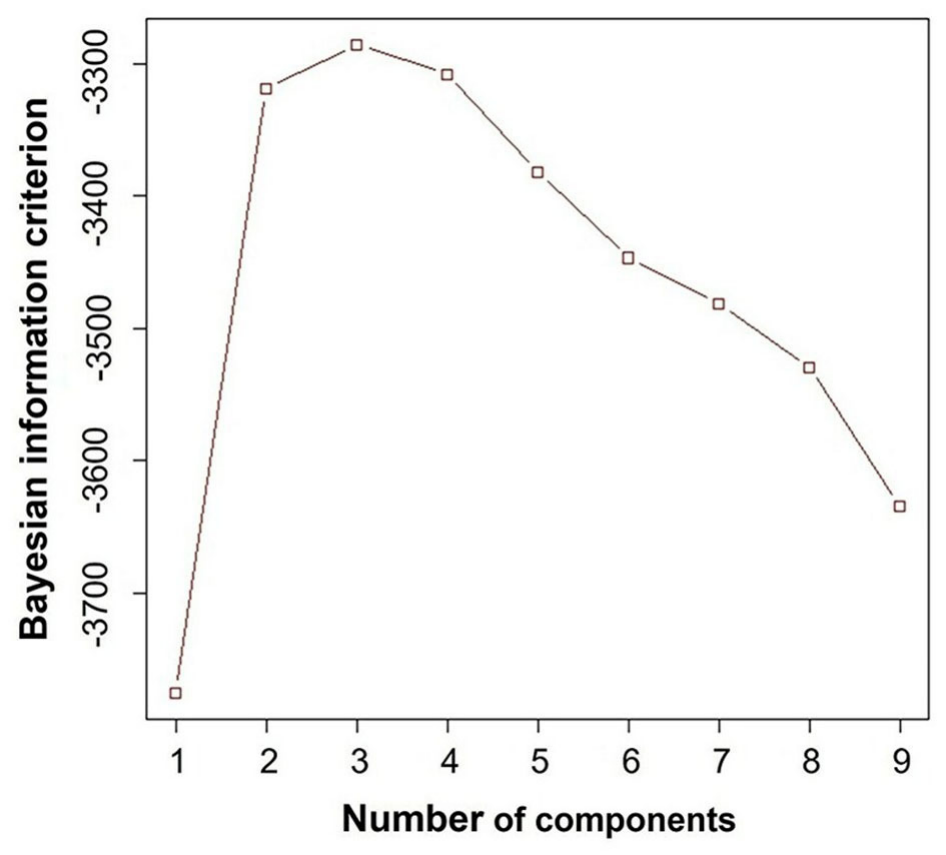
Bayesian information criterion. This result demonstrated that three-cluster model fit the best, because the absolute value of Bayesian information criterion was the lowest when the dataset was modeled with three clusters.

After confirming that diastolic variables (E/A, e', E/e', LAVi and TRV) were not strongly correlated each other (Table 2), we performed a cluster analysis using only these variables as input. Bayesian information criterion indicated that a three-cluster models best fit the dataset, because absolute value of Bayesian information criterion was the lowest when the dataset was modeled with three clusters (Figure 3).

**Table 2:** Partial correlations among diastolic function variables.

**E/A**	**e'**	**E/e'**	**LAVi**	**TRV**	
1	0.03	0.41	0.46	0.53	E/A
	1	−0.55	−0.25	−0.10	e'
		1	0.45	0.44	E/e'
			1	0.46	LAVi
				1	TRV

*Abbreviations were the same as in Table 1*.

E/e' (*p* < 0.001), LAVi (*p* < 0.001) and TRV (*p* < 0.001) progressively increased from cluster 1 to 3, as assigned (Table 3, Figure 2). Cluster 1 had higher E/A and e' than cluster 2, indicating that cluster 1 represented less abnormal diastolic function. From cluster 1 to 3, patients were older (*p* < 0.001) and thinner (*p* = 0.028). HFpEF was dominant in clusters 1 (59%) and 2 (50%), whereas HFrEF was more prevalent in cluster 3 (44%). LV myocardial function measured by LVGLS and LV circumferential strains was abnormal in all the clusters and decreased gradually from cluster 1 to 3 (*p* = 0.018 and *p* = 0.005, respectively). Right ventricular function was reduced in cluster 3 (*p* = 0.010). These trends resulted in increasing MAGGIC scores from cluster 1 to 3 (*p* < 0.001).

**Table 3:** Comparison of baseline characteristics by clusters.

	**Cluster**			
	**Cluster 1** **(*n* = 46)**	**Cluster 2** **(*n* = 167)**	**Cluster 3** **(*n* = 66)**	***P*** **value**
Age, years	60 ± 16	68 ± 13	71 ± 15	<0.001
Male gender, *n* (%)	34 (74)	118 (71)	41 (62)	0.333
BMI, kg/m^2^	24.3 ± 5.8	24.3 ± 5.1	22.4 ± 4.4	0.028
SBP, mmHg	116 ± 20	123 ± 18	116 ± 20	0.008
HR, beat/min	67 ± 12	66 ± 11	70 ± 14	0.127
**Comorbidities**				
Hypertension, *n* (%)	26 (57)	106 (64)	51 (77)	0.050
Diabetes, *n* (%)	14 (30)	65 (39)	24 (36)	0.569
**Underlying heart disease**				
CAD, *n* (%)	14 (30)	56 (34)	26 (39)	0.575
Paroxysmal AF, *n* (%)	12 (26)	54 (32)	25 (38)	0.421
HF duration, years	1.1 ± 1.8	1.5 ± 2.9	1.5 ± 2.5	0.716
MAGGIC score	19 ± 8	21 ± 8	26 ± 8	<0.001
**Medications**				
ACEi, *n* (%)	13 (28)	55 (33)	18 (27)	0.644
ARB, *n* (%)	22 (48)	86 (52)	34 (52)	0.901
β-blocker, *n* (%)	32 (70)	131 (78)	48 (73)	0.380
Loop diuretics, *n* (%)	29 (63)	114 (68)	54 (82)	0.058
MRA, *n* (%)	21 (46)	66 (40)	27 (41)	0.755
**Devices**				
Pacemaker, *n* (%)	1 (2)	12 (7)	6 (9)	0.344
CRT, *n* (%)	0 (0)	3 (2)	0 (0)	0.362
ICD, *n* (%)	1 (2)	8 (5)	1 (2)	0.410
**Laboratory**				
Albumin, g/dl	4.1 ± 0.4	4.0 ± 0.5	3.7 ± 0.4	<0.001
Hemoglobin, g/dl	13.7 ± 2.1	13.3 ± 1.9	12.6 ± 2.3	0.017
Creatinine, mol/l	89 ± 39	97 ± 44	101 ± 35	0.318
eGFR, ml/min/1.73m^2^	65.4 ± 22.6	55.3 ± 20.1	50.8 ± 18.7	0.001
BNP, pg/ml	113 ± 159	137 ± 163	547 ± 659	<0.001
**Electrocardiography**				
QRS duration, msec	102 ± 20	116 ± 39	115 ± 30	0.081
**Echocardiography**				
LVEDV, ml	133 ± 61	131 ± 63	147 ± 76	0.256
LVMi, g/m^2^	133 ± 61	155 ± 46	181 ± 69	<0.001
RWT	0.35 ± 0.10	0.37 ± 0.10	0.35 ± 0.14	0.452
LVEF, %	51 ± 19	50 ± 16	44 ± 16	0.018
<40%, *n* (%)	13 (28)	51 (31)	29 (44)	0.105
40–49%, *n* (%)	6 (13)	32 (19)	14 (21)	
≥ 50%, *n* (%)	27 (59)	84 (50)	23 (35)	
GLS, %	−12.1 ± 4.6	−11.0 ± 4.4	−9.6 ± 5.4	0.018
GCS, %	−19.4 ± 8.3	−17.0 ± 8.5	−14.4 ± 7.2	0.005
s', cm/sec	5.7 ± 1.7	4.9 ± 1.5	4.2 ± 1.4	<0.001
e', cm/sec	6.1 ± 1.7	4.0 ± 1.1	4.2 ± 1.4	<0.001
a', cm/sec	7.2 ± 2.3	6.8 ± 1.8	4.5 ± 1.7	<0.001
E/A	1.19 ± 0.39	0.75 ± 0.23	2.24 ± 1.33	<0.001
E, cm/s	63 ± 13	54 ± 16	88 ± 25	<0.001
E/e'	11.0 ± 3.4	14.0 ± 4.9	25.0 ± 10.6	<0.001
LAVi, ml/m^2^	26.3 ± 8.6	30.9 ± 12.8	56.6 ± 15.9	<0.001
TRV, m/sec	2.2 ± 0.3	2.2 ± 0.3	2.8 ± 0.5	<0.001
RVEDA, cm^2^	15.8 ± 5.2	15.5 ± 4.9	17.5 ± 6.6	0.038
RVFAC, %	50 ± 13	49 ± 13	43 ± 16	0.010

*Abbreviations were the same as in Table 1*.

### Clinical Relevance

**Figure 4:**
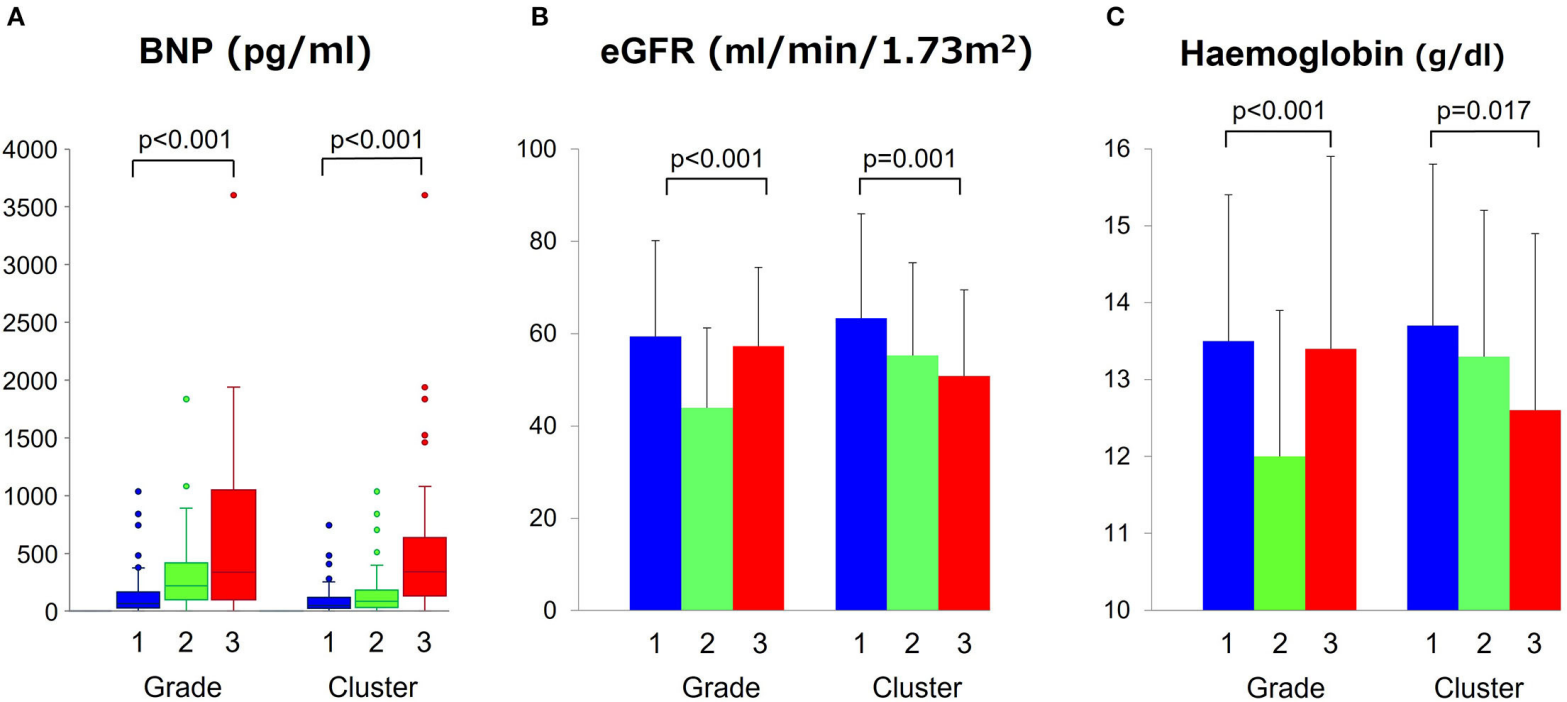
Comparisons of BNP, eGFR and hemoglobin level across grades and clusters. **(A)** Comparisons of BNP, **(B)** eGFR, and **(C)** hemoglobin level across grades and clusters. BNP, brain natriuretic peptide; eGFR, estimate glomerular filtration rate.

Figure 4A compares BNP level across the clusters and grades; in 193 patients (70%) in whom BNP measurements were available, the median values increased similarly for both classifications (*p* < 0.001). There were no significant differences in major baseline characteristics between patients with and without BNP measurement (Table 4). Figures 4B,C show progressive anemia (*p* = 0.017) and worsening renal function (eGFR, *p* = 0.001) across the clusters but not across the grades.

**Table 4:** Comparison of baseline characteristics between patients with and without BNP measurement.

	**Patients with** **BNP measurement** **(*n* = 193)**	**Patients without** **BNP measurement** **(*n* = 86)**	***P*** **value**
Age, years	68 ± 15	67 ± 14	0.504
Male gender, *n* (%)	131 (68)	62 (72)	0.481
BMI, kg/m^2^	24.0 ± 5.3	23.5 ± 4.6	0.404
SBP, mmHg	121 ± 20	119 ± 18	0.558
HR, beat/min	67 ± 12	68 ± 13	0.612
**Comorbidities**			
Hypertension, *n* (%)	129 (67)	54 (63)	0.511
Diabetes, *n* (%)	68 (35)	35 (41)	0.382
**Underlying heart disease**			
Paroxysmal AF, *n* (%)	68 (35)	13 (27)	0.163
CAD, *n* (%)	63 (33)	33 (11)	<0.001
HF duration, years	1.2 ± 2.5	1.9 ± 2.9	0.028
MAGGIC score	22 ± 8	22 ± 8	0.649
**Medications**			
ACEi, *n* (%)	56 (29)	30 (35)	0.327
ARB, *n* (%)	102 (53)	40 (47)	0.328
β-blocker, *n* (%)	139 (72)	72 (84)	0.036
Loop diuretics, *n* (%)	142 (74)	55 (64)	0.103
MRA, *n* (%)	80 (42)	34 (40)	0.764
**Devices**			
Pacemaker, *n* (%)	14 (7)	5 (6)	0.659
CRT, *n* (%)	3 (2)	0 (0)	0.245
ICD, *n* (%)	9 (5)	1 (1)	0.146
**Laboratory**			
Albumin, g/dl	3.9 ± 0.5	3.9 ± 0.5	0.849
Hemoglobin, g/dl	13.1 ± 2.0	13.4 ± 2.2	0.234
Creatinine, mol/l	97 ± 44	97 ± 35	0.432
eGFR, ml/min/1.73m^2^	55.2 ± 21.0	57.3 ± 19.8	0.440
**Electrocardiography**			
QRS duration, msec	113 ± 37	113 ± 28	0.967
**Echocardiography**			
LVEDV, ml	132 ± 64	144 ± 71	0.152
LVMi, g/m^2^	156 ± 49	160 ± 66	0.634
RWT	0.37 ± 0.11	0.34 ± 0.11	0.044
LVEF, %	50 ± 16	46 ± 17	0.059
GLS, %	−11.1 ± 4.8	−10.3 ± 4.5	0.228
GCS, %	−17.3 ± 8.2	−15.6 ± 8.5	0.114
s', cm/sec	4.9 ± 1.6	4.8 ± 1.7	0.441
e', cm/sec	4.4 ± 1.4	4.3 ± 1.6	0.563
a', cm/sec	6.4 ± 2.1	6.3 ± 2.3	0.757
E/A	1.18 ± 0.93	1.16 ± 0.90	0.863
E, cm/s	64 ± 23	61 ± 24	0.392
E/e'	16.1 ± 7.8	16.2 ± 9.3	0.919
LAVi, ml/m^2^	36.5 ± 17.7	35.6 ± 16.7	0.975
TRV, m/sec	2.3 ± 0.4	2.3 ± 0.4	0.526
RVEDA, cm^2^	16 ± 6	16 ± 4	0.713
RVFAC, %	49 ± 14	46 ± 14	0.194

*Abbreviations were the same as in Table 1*.

**Figure 5:**
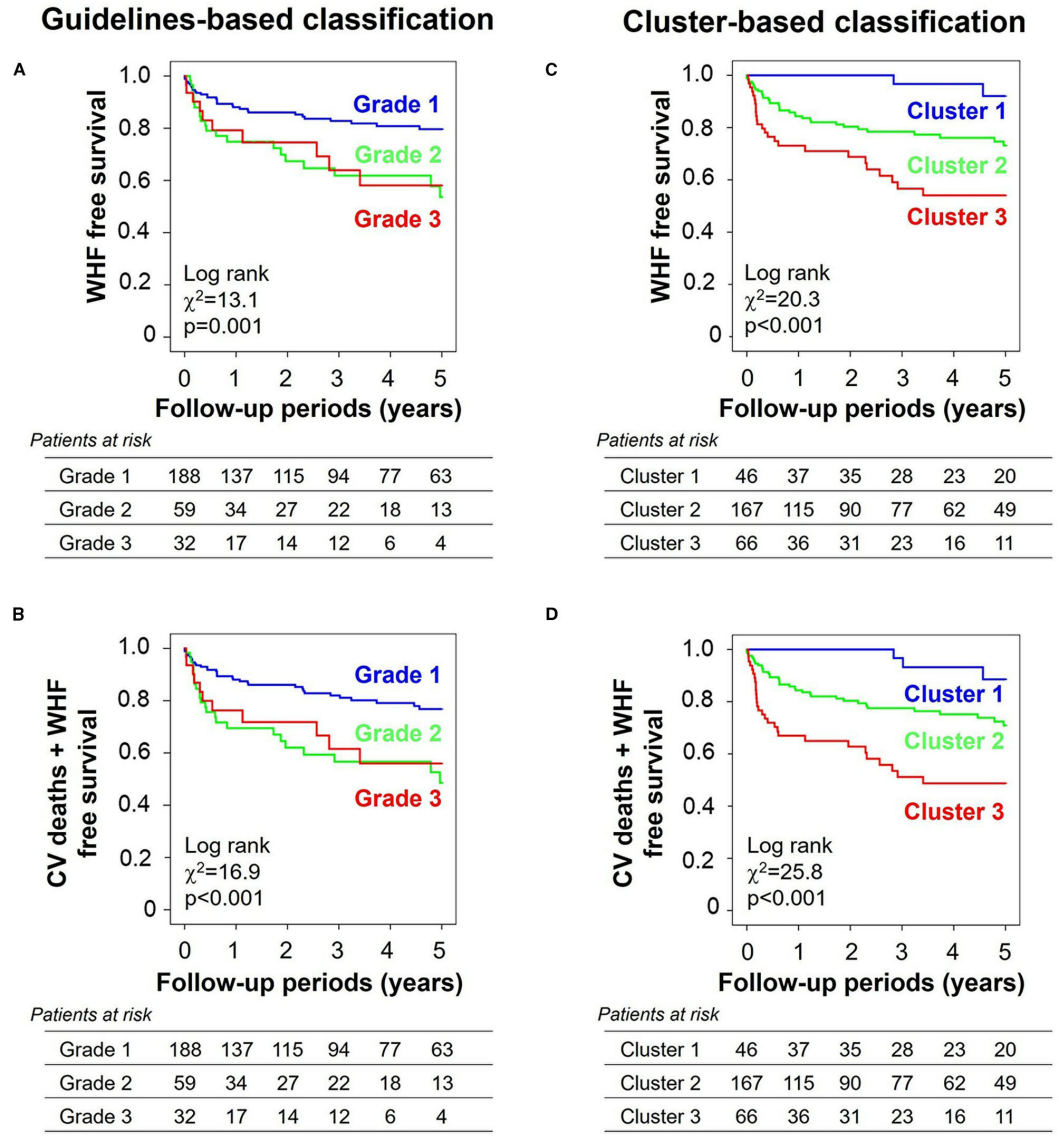
Kaplan–Meier curves stratified by grades and clusters. **(A)** Primary endpoint (WHF) and **(B)** secondary endpoint (a composite of CV deaths and WHF) when stratified by guidelines-based classification. **(C)** Primary endpoint and **(D)** secondary endpoint when stratified by cluster-based classification. WHF, worsening heart failure; CV, cardiovascular.

**Figure 6:**
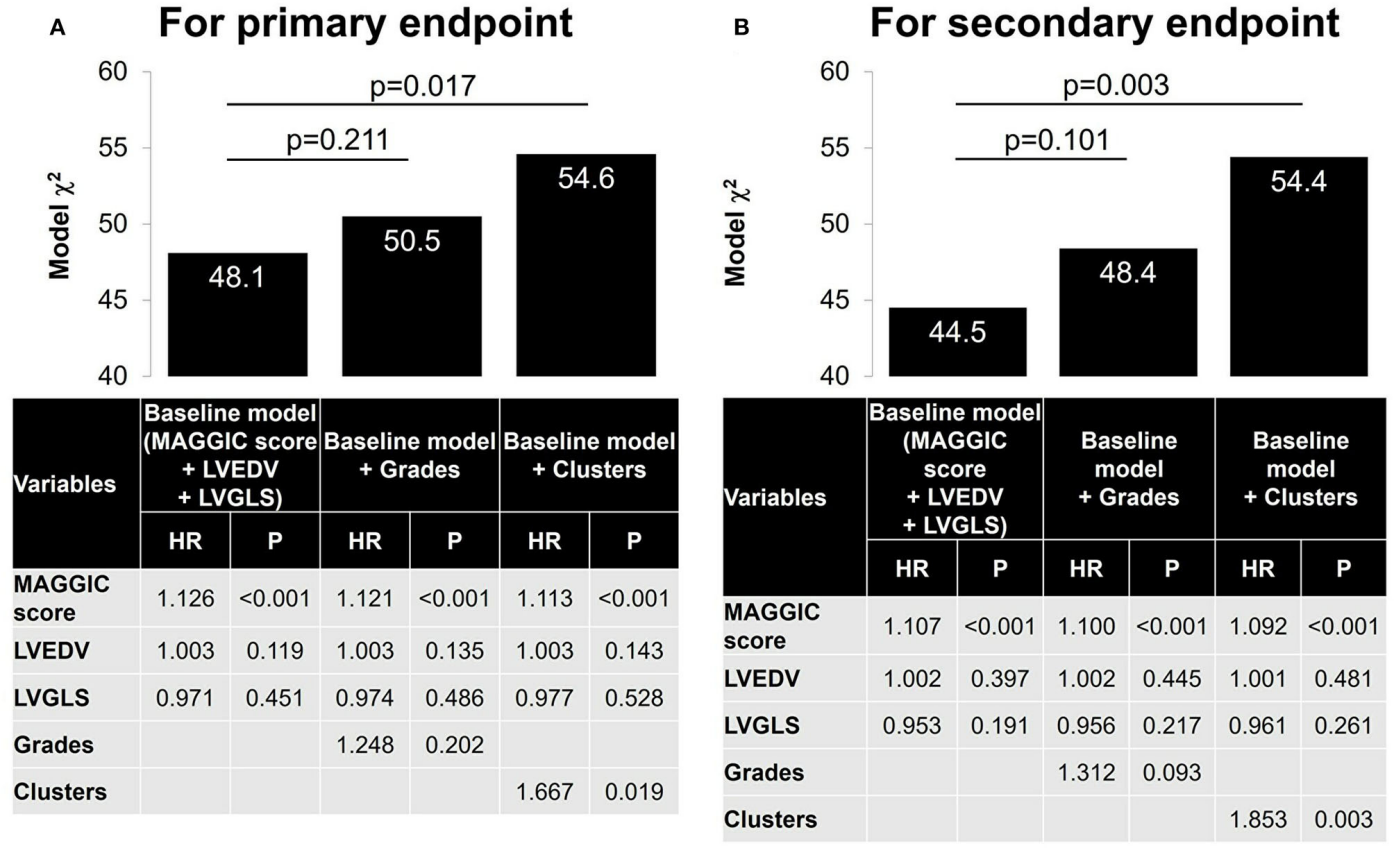
Nested Cox models. A baseline Cox model was first constructed with MAGGIC score, LVEDV and LVGLS. A nested model was then constructed by adding grades and cluster separately. **(A)** For primary endpoint. **(B)** For secondary endpoint. MAGGIC, Meta-analysis Global Group in Chronic Heart Failure; LVEDV, left ventricular end-diastolic volume; LVGLS, left ventricular global longitudinal strain.

During a follow-up period of 2.6 ± 2.0 years, 62 patients (22%) experienced the primary endpoint of worsening HF, and 69 patients (25%) experienced the secondary composite endpoint of worsening HF (62 patients) and/or cardiovascular death (seven patients). Figure 5 compares survival curves stratified by guidelines-based classification with those obtained using cluster-based classification. When stratified by grades, the survival curves showed significant overall separations for both primary and secondary endpoints, but grades 2 and 3 diastolic dysfunction had similar survival curves (Figures 5A,B). Cluster-based classification, however, produced clearer separations of survival curves for both primary and secondary endpoints (Figures 5C,D), as demonstrated by higher χ^2^ (primary endpoint: χ^2^ = 20.3, *p* < 0.001 for clusters, χ^2^ = 13.1, *p* = 0.001 for grades; secondary endpoint: χ^2^ = 25.8, *p* < 0.001 for clusters, χ^2^ = 16.9, *p* < 0.001 for grades). In the sequential Cox proportional hazard analysis, the baseline model including MAGGIC score, LVEDV and LVGLS gave an overall χ^2^ value of 48.1 for the primary endpoint and 44.5 for the secondary endpoint (Figure 6). The addition of diastolic function grades did not improve the predictive power (χ^2^ = 50.5, *p* = 0.211 for primary endpoint; χ^2^ = 48.4, *p* = 0.101 for secondary endpoint), whereas the addition of clusters significantly improved the predictive power for both study endpoints (χ^2^ = 54.6, *p* = 0.017 for primary endpoint: and χ^2^ = 54.4, *p* = 0.003 for secondary endpoint).

### Concordance Between Cluster-Based and Guidelines-Based Classifications

**Figure 7:**
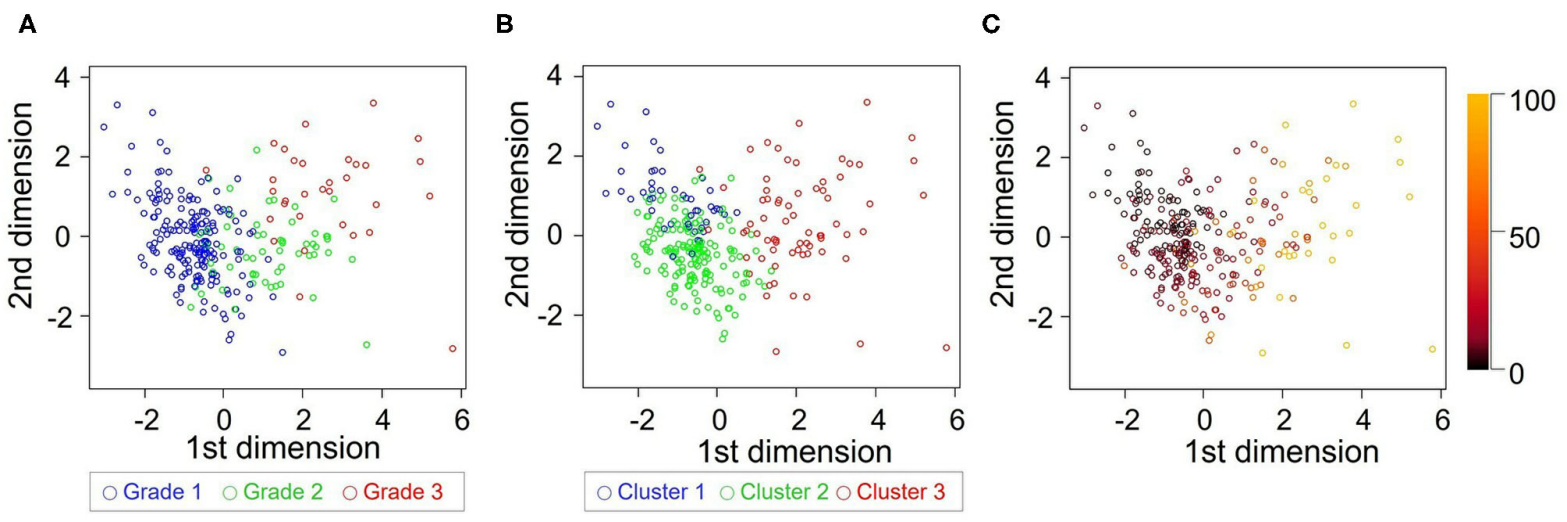
Comparison of grade and cluster distribution. Grade **(A)** and cluster **(B)** distribution in the first 2 dimensions identified by principal component analysis. **(C)** Mahalanobis distance from each subject to the center of cluster 1 was color-coded.

The patterns of grade and cluster distributions were mapped in Figure 7. The whole study population was distributed in a U-shape; grade 1 to 3 were aligned sequentially from the left to the right side, indicating that there was a spatial gradient of diastolic function in the patient distribution map; cluster 1–3 were aligned similarly, although the boundaries between the clusters shifted leftward. Table 5 shows a contingency table comparing cluster-based classification against guidelines-based classification; moderate ordinal association was observed (Kappa statistic = 0.113, Kendall's correlation coefficient = 0.599, *p* < 0.001 for both statistics). Patients diagnosed as grade 1 diastolic dysfunction by the guidelines (n = 188) were allocated mostly to cluster 1 (22%) and cluster 2 (76%) but 5 patients (3%) were allocated even to cluster 3.

**Table 5:** Concordance between cluster-based and guidelines-based classifications.

	**Cluster 1**	**Cluster 2**	**Cluster 3**	**Total**
Grade 1	41 (22%)	142 (76%)	5 (3%)	188
Grade 2	4 (7%)	25 (42%)	30 (51%)	59
Grade 3	1 (3%)	0 (0%)	31 (97%)	32
Total	46	167	66	279

**Figure 8:**
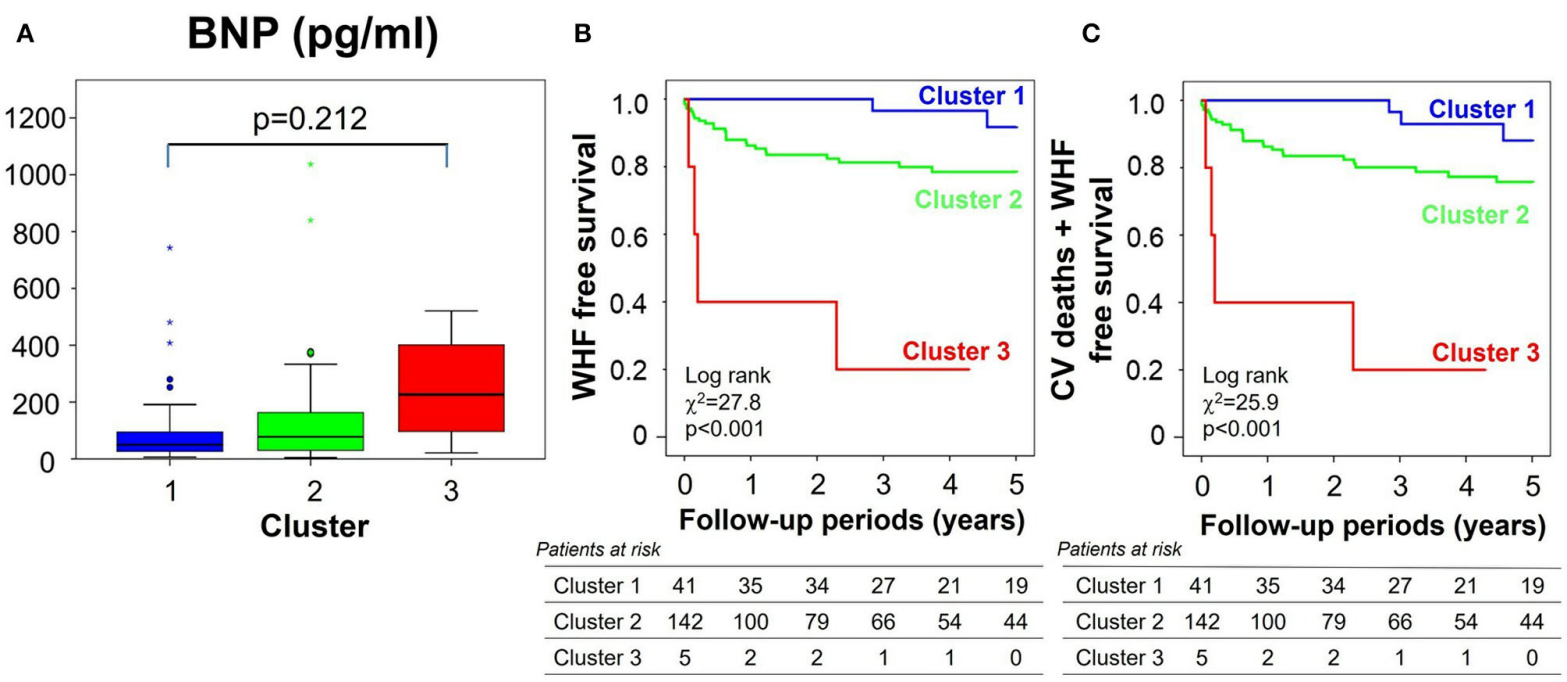
Clinical validations of clusters in the subgroup of all 188 patients with grade 1 diastolic dysfunction by echocardiographic criteria. **(A)** Comparison of BNP level, **(B,C)** clinical outcomes [**(B)** for primary endpoint, **(C)** for secondary endpoint] stratified by clusters. BNP, brain natriuretic peptide; WHF, worsening heart failure; CV, cardiovascular.

To assess whether clustering helped stratify risk in grade 1 diastolic dysfunction, BNP levels and clinical outcomes were assessed within this subgroup (Figure 8). BNP levels increased from cluster 1 to cluster 3, but not significantly so perhaps because of the small number of subjects in cluster 3 in this subgroup. Kaplan-Meier curves demonstrated a progressive deterioration in prognosis for both primary and secondary endpoints from cluster 1–3, that was significant by Cox regression analysis (hazard ratio = 5.61, *p* < 0.001) even after adjusting for MAGGIC score, LVEDV and LVGLS.

## Discussion

We demonstrated that cluster analysis outperforms diastolic function classification by echocardiographic consensus recommendations, for the prediction of hospitalizations or cardiovascular deaths in patients with HF, irrespective of LVEF. The improved performance was the greatest for subjects with grade 1 diastolic dysfunction. These results suggest a more data-driven approach for developing diagnostic recommendations.

### Advantage of Using Cluster Analysis for Discriminating Diastolic Function Patterns

Precise assessment of diastolic function is essential for diagnosing and managing HF. To grade diastolic function in clinical practice, the consensus recommendations have proposed complex decision-tree algorithms that evolved through multiple iterations (7). The update published in 2016 narrowed down the diastolic variables and simplified the algorithms for ease of daily clinical application. The iterations resulted in considerable changes in the diagnosis of diastolic function, as shown by retrospective analyses of population-based cohorts; (13, 14) it decreased when diagnosed by the 2016 recommendation (1.4%), compared with the 2009 recommendations (38.1%) (13). Large validation studies indicated that the 2016 update increased specificity for detecting elevated LV filling pressure (from 70–75 to 74–81%) but did not improve overall accuracy (67–75%, compared to 63–74% for the 2009 version) (15–17).

There are some reasons why the current diagnostic algorithms remain suboptimal. The algorithms have been developed by theoretical considerations that diastolic variables uniformly follow typical changes during the progression from normal to severe diastolic dysfunction in all patients (1, 2). However, LV diastolic filling involves several distinct physiological processes, including myocardial relaxation and LV compliance (7). These processes may be differentially affected in different cardiac diseases (18, 19). Because each diastolic variable reflects a different aspect of LV diastolic filling, it would be better if diastolic variables are considered separately and independently.

The current diagnostic algorithms use discrete categorizations defined by cut-off points. These categorizations are easier for us to analyze and interpret than measured continuous variables themselves, but they cause a loss of information on between-subject variability. For example, two subjects between whom a diastolic variable differs greatly but with both values above the cut-off point will be graded similarly, while two subjects with a similar difference in a diastolic variable but with one value lying above and the other below the cut-off point will be categorized in different grades. Echocardiographic measurements of diastolic variables are subject to errors of 5–10% (20). This small degree of variance can result in different grading, if measured variables fall close to cut-off points. Each diastolic variable has been shown to correlate with left atrial pressure (15), so treating them as continuous would be better than dichotomizing them to predict left atrial pressure.

Cluster analysis captures the natural structure of multivariate data without a priori knowledge and it has been applied extensively in medical science, for example to identify clinical phenotypes (8, 21). This approach is suitable for creating new diagnostic criteria for LV diastolic function, because it can overcome the above issues involved in the current diagnostic recommendations. It can categorize diastolic function, independent of the current diagnostic labels, so that the resulting model will not be biased by possible erroneous diagnoses. It can also treat multiple variables independently and continuous variables as continuous.

A previous study has already investigated the natural clustering of diastolic variables and successfully isolated a high-risk phenotype in a convenience sample of subjects predominantly with preserved LVEF (22). Our current study extended this application to a consecutive series of patients with HF comprised equally of HFpEF and HFrEF and confirmed that cluster analysis blindly identified distinct diastolic function phenotypes that exhibited a clear association with BNP level and a more significant prognostic impact than did conventional grading of diastolic function. The learned clusters were found to correlate with LV myocardial strains and with the prevalence of cardiovascular risk factors such as age, hypertension and chronic kidney disease, more than did the diastolic function grades. These correlations may lead to an increased sensitivity for detecting at-risk patients, especially when diastolic function was diagnosed as grade 1 by the consensus recommendations.

### Clinical Implications

The learned clusters can be used for assessing new patients. Model-based cluster analysis, which we used in this study, will give the probability of membership for each cluster or distance from each cluster if we enter diastolic variables obtained from new patients into the learned model (Figure 7C); those criteria could then be used to help diagnose HF or quantify treatment effects. A prognostic score developed using cluster analysis can also be more adaptable and give a continuous probability of adverse outcomes in HF. Large-scale datasets of echocardiographic diastolic variables are required to establish a definitive cluster model.

There are several limitations. Firstly, because of the retrospective nature of this study, there were some patients in the HF database in whom all the diastolic variables were not measured. We excluded them from the study population to avoid imputation for cluster modeling. This exclusion may bias the results. Secondly, we did not include stress echocardiographic data in the cluster modeling. Previous studies have demonstrated that diastolic variables obtained using resting echocardiography were not sensitive enough to diagnose elevated left atrial pressure and diastolic stress test would improve the diagnosis (23), so current consensus recommendations advise the use of stress tests to diagnose HF, especially when LVEF is preserved (24). However, a major purpose of this study was to examine whether cluster analysis would improve the diagnosis of diastolic function over current diagnostic criteria, so we included those diastolic variables measured at rest that are listed in the current recommendations. Thirdly, we studied patients with preserved and reduced LVEFs together. The diagnostic accuracy of the current diagnostic criteria has been shown to depend on LVEF (16), so the results might be different if diastolic variables were modeled separately for preserved and reduced LVEFs. Fourthly, septal E/e' instead of average E/e' was used for diastolic grading and clustering, because measuring e' at the septum was our standard protocol at that time. We acknowledge that E/e' is recommended to be measured by averaging septal and lateral e' and lateral e' has been shown to be more sensitive for detecting diastolic dysfunction (25). Fifth, patients with atrial fibrillation, which commonly coexists in HF, were excluded in this study, because their diastolic function cannot be graded by the current diagnostic algorithm. Diastolic variables used for grading in sinus rhythm cannot always be measured nor are useful in atrial fibrillation, for example, E/A and LAVi. Separate clustering would be required to model diastolic function in atrial fibrillation. Sixth, our learned model has not been tested in a different population. External validation is required to assess the generalizability of the model.

## Conclusions

Machine learning allows echocardiographic diastolic function phenotyping that associates with HF biomarker and stratifies HF risk better than the current recommendations. The results of this study provide the basis for applying this data-driven approach for precise diagnosis and prognostication in HF, in order to formulate diagnostic recommendations that are based on evidence rather than consensus.

## Data Availability Statement

The original contributions presented in the study are included in the article/Supplementary Material, further inquiries can be directed to the corresponding author.

## Author Contributions

HK and TU contributed to conception and design of the study, performed statistical analyses, interpreted results, and drafted the manuscript. HK, TU, LT, JC, SS, and YK constructed the database. AF helped revise the manuscript. All authors approved the final version of the manuscript.

## Conflict of Interest

TU received a research funding from Hitachi. SS received a lecture fee from Daiichi-Sankyo and a research funding from Daiichi-Sankyo and Mitsubishi-Tanabe. TY has received a research funding from Daiichi-Sankyo, Bayer Healthcare, and Bristol Meyers Squibb and a remuneration from Daiichi-Sankyo, Pfizer, Bayer Healthcare, Bristol-Myers Squibb, Toa Eiyo, and Ono Pharmaceutical. The remaining authors declare that the research was conducted in the absence of any commercial or financial relationships that could be construed as a potential conflict of interest.

## Publisher's Note

All claims expressed in this article are solely those of the authors and do not necessarily represent those of their affiliated organizations, or those of the publisher, the editors and the reviewers. Any product that may be evaluated in this article, or claim that may be made by its manufacturer, is not guaranteed or endorsed by the publisher.

## References

[1] Redfield MM Jacobsen SJ Burnett JC Jr Mahoney DW Bailey KR Rodeheffer RJ. Burden of systolic and diastolic ventricular dysfunction in the community: appreciating the scope of the heart failure epidemic. JAMA. (2003) 289:194–202. 10.1001/jama.289.2.194 12517230

[2] Nagueh SF. Left ventricular diastolic function: understanding pathophysiology, diagnosis, and prognosis with echocardiography. JACC Cardiovasc Imaging. (2020) 13:228–44. 10.1016/j.jcmg.2018.10.038 30982669

[3] Somaratne JB Whalley GA Gamble GD Doughty RN. Restrictive filling pattern is a powerful predictor of heart failure events postacute myocardial infarction and in established heart failure: a literature-based meta-analysis. J Card Fail. (2007) 13:346–52. 10.1016/j.cardfail.2007.01.010 17602980

[4] Wang M Yip GWK Wang AYM Zhang Y Ho PY Tse MK. Peak early diastolic mitral annulus velocity by tissue Doppler imaging adds independent and incremental prognostic value. J Am Coll Cardiol. (2003) 41:820–6. 10.1016/S0735-1097(02)02921-2 12628728

[5] Hillis GS Møller JE Pellikka PA Gersh BJ Wright RS Ommen SR. Noninvasive estimation of left ventricular filling pressure by E/e' is a powerful predictor of survival after acute myocardial infarction. J Am Coll Cardiol. (2004) 43:360–7. 10.1016/j.jacc.2003.07.044 15013115

[6] Nagueh SF Bhatt R Vivo RP Krim SR Sarvari SI Russell K. Echocardiographic evaluation of hemodynamics in patients with decompensated systolic heart failure. Circ Cardiovasc Imaging. (2011) 4:220–7. 10.1161/CIRCIMAGING.111.963496 21398512

[7] Nagueh SF Smiseth OA Appleton CP Byrd BF 3rd Dokainish H Edvardsen T. Recommendations for the evaluation of left ventricular diastolic function by echocardiography: an update from the American society of echocardiography and the european association of cardiovascular imaging. J Am Soc Echocardiogr. (2016) 29:277–314. 10.1016/j.echo.2016.01.011 27037982

[8] Ahmad T Pencina MJ Schulte PJ O'Brien E Whellan DJ Pina IL. Clinical implications of chronic heart failure phenotypes defined by cluster analysis. J Am Coll Cardiol. (2014) 64:1765–74. 10.1016/j.jacc.2014.07.979 25443696 PMC4254424

[9] Shah SJ Katz DH Selvaraj S Burke MA Yancy CW Gheorghiade M. Phenomapping for novel classification of heart failure with preserved ejection fraction. Circulation. (2015) 131:269–79. 10.1161/CIRCULATIONAHA.114.010637 25398313 PMC4302027

[10] Cikes M Sanchez-Martinez S Claggett B Duchateau N Piella G Butakoff C. Machine learning-based phenogrouping in heart failure to identify responders to cardiac resynchronization therapy. Eur J Heart Fail. (2019) 21:74–85. 10.1002/ejhf.1333 30328654

[11] Fraley C Raftery AE. Model-based clustering, discriminant analysis and density estimation. J Am Stat Assoc. (2002) 97:611–31. 10.1198/016214502760047131

[12] Pocock SJ Ariti CA McMurray JJ Maggioni A Køber L Squire IB. Predicting survival in heart failure: a risk score based on 39 372 patients from 30 studies. Eur Heart J. (2013) 34:1404–13. 10.1093/eurheartj/ehs337 23095984

[13] Almeida JG Fontes-Carvalho R Sampaio F Ribeiro J Bettencourt P Flachskampf FA. Impact of the 2016 ASE/EACVI recommendations on the prevalence of diastolic dysfunction in the general population. Eur Heart J Cardiovasc Imaging. (2018) 19:380–6. 10.1093/ehjci/jex252 29236978

[14] Huttin O Fraser AG Coiro S Bozec E Selton-Suty C Lamiral Z. Impact of changes in consensus diagnostic recommendations on the echocardiographic prevalence of diastolic dysfunction. J Am Coll Cardiol. (2017) 69:3119–21. 10.1016/j.jacc.2017.04.039 28641802

[15] Balaney B Medvedofsky D Mediratta A Singh A Ciszek B Kruse E. Invasive Validation of the echocardiographic assessment of left ventricular filling pressures using the 2016 diastolic guidelines: head-to-head comparison with the 2009 guidelines. J Am Soc Echocardiogr. (2018) 31:79–88. 10.1016/j.echo.2017.09.002 29111121 PMC5756671

[16] Andersen OS Smiseth OA Dokainish H Abudiab MM Schutt RC Kumar A. Estimating left ventricular filling pressure by echocardiography. J Am Coll Cardiol. (2017) 69:1937–48. 10.1016/j.jacc.2017.01.058 28408024

[17] Lancellotti P Galderisi M Edvardsen T Donal E Goliasch G Cardim N. Echo-Doppler estimation of left ventricular filling pressure: results of the multicentre EACVI Euro-Filling study. Eur Heart J Cardiovasc Imaging. (2017) 18:961–8. 10.1093/ehjci/jex067 28444160

[18] Nishimura RA Appleton CP Redfield MM Ilstrup DM Holmes DR Jr Tajik AJ. Noninvasive doppler echocardiographic evaluation of left ventricular filling pressures in patients with cardiomyopathies: a simultaneous Doppler echocardiographic and cardiac catheterization study. J Am Coll Cardiol. (1996) 28:1226–33. 10.1016/S0735-1097(96)00315-4 8890820

[19] Rakowski H Carasso S. Quantifying diastolic function in hypertrophic cardiomyopathy: the ongoing search for the holy grail. Circulation. (2007) 116:2662–5. 10.1161/CIRCULATIONAHA.107.742395 18056537

[20] Spirito P Maron BJ Verter I Merrill JS. Reproducibility of Doppler echocardiographic measurements of left ventricular diastolic function. Eur Heart J. (1988) 9:879–86. 10.1093/oxfordjournals.eurheartj.a062582 3181174

[21] Sanchez-Martinez S Duchateau N Erdei T Kunszt G Aakhus S Degiovanni A. Machine learning analysis of left ventricular function to characterize heart failure with preserved ejection fraction. Circ Cardiovasc Imaging. (2018) 11:e007138. 10.1161/CIRCIMAGING.117.007138 29661795

[22] Lancaster MC Salem Omar AM Narula S Kulkarni H Narula J Sengupta PP. Phenotypic clustering of left ventricular diastolic function parameters: patterns and prognostic relevance. JACC Cardiovasc Imaging. (2019) 12:1149–61. 10.1016/j.jcmg.2018.02.005 29680357

[23] Obokata M Kane GC Reddy YN Olson TP Melenovsky V Borlaug BA. Role of diastolic stress testing in the evaluation for heart failure with preserved ejection fraction: a simultaneous invasive-echocardiographic study. Circulation. (2017) 135:825–38. 10.1161/CIRCULATIONAHA.116.024822 28039229 PMC5330848

[24] Pieske B Tschöpe C de Boer RA Fraser AG Anker SD Donal E. How to diagnose heart failure with preserved ejection fraction: the HFA-PEFF diagnostic algorithm: a consensus recommendation from the Heart Failure Association (HFA) of the European Society of Cardiology (ESC). Eur J Heart Fail. (2020) 22:391–412. 10.1002/ejhf.1741 32133741

[25] Arques S Roux E Sbragia P Ambrosi P Taieb L Pieri B. Accuracy of tissue Doppler echocardiography in the emergency diagnosis of decompensated heart failure with preserved left ventricular systolic function: comparison with B-type natriuretic peptide measurement. Echocardiography. (2005) 22:657–64. 10.1111/j.1540-8175.2005.40076.x 16174119

