# Supplementary material for: A Phenotyping of Diastolic Function by Machine Learning Improves Prediction of Clinical Outcomes in Heart Failure
Source: Front Cardiovasc Med. 2021 Dec 23;8:755109. doi: 10.3389/fcvm.2021.755109 (PMC8733156; doi:10.3389/fcvm.2021.755109)
Supplement: Supplementary file 1 [file Table_1.DOCX]

Supplementary Material

# Supplemental method

**Cluster analysis**

In this study, for phenotyping diastolic properties, a model-based cluster analysis was performed on five diastolic variables (E/A, e’, E/e’, LAVi and TRV) as listed in the current consensus recommendation, because it has been successfully applied in medical science for phenotyping diseases. It models the distribution of study subjects with a mixture of a prespecified number of gaussian distributions. Each gaussian distribution represents one cluster. The optimal number of clusters was selected based on Bayesian information criterion. The absolute value of Bayesian information criterion becomes the lowest when the number of clusters is optimal. Before modelling, it was confirmed that the variables were not significantly correlated each other by partial correlation analysis. All the variables were standardized to a mean = 0 and a standard deviation = 1 so that they were equally weighted in the analysis. For modelling, the model parameters were estimated using expectation-maximization algorithm. Once the gaussian mixture model was created, for each subject, the probability of membership for each cluster was calculated. The subject was assigned to a cluster where the probability of membership was the highest. The above cluster analysis was performed using mclust package in R (version 3.5.1, Vienna, Austria). This software package provides a comprehensive analysis covering density estimation of dataset, model fitting and clustering.
